# Non-invasive prediction of the first ventilatory threshold in Chinese patients with chronic heart failure for personalized exercise prescription

**DOI:** 10.3389/fcvm.2026.1805304

**Published:** 2026-07-06

**Authors:** Xiaoling Liu, Jue Yuan, Qingxuan Yang, Yuxuan Fan, Wenjuan Xiu, Yuqin Shen

**Affiliations:** 1 Department of Rehabilitation, Tongji Hospital Affiliated to Tongji University, School of Medicine, Tongji University, Shanghai, China; 2 Department of Geriatrics/Geriatric Rehabilitation, Shanghai Yangzhi Rehabilitation Hospital (Shanghai Sunshine Rehabilitation Center), School of Medicine, Tongji University, Shanghai, China

**Keywords:** aerobic exercise, cardiac rehabilitation, cardiopulmonary exercise test, first ventilatory threshold, prediction model

## Abstract

**Purpose:**

This study aimed to develop non-invasive prediction models for the first ventilatory threshold (VT_1_) in patients with chronic heart failure (CHF), to address the inaccuracy of guideline-recommended, percentage-based exercise intensity prescriptions (40%–69% peak VO_2_, 55%–75% peak HR) and the limited accessibility of gold-standard cardiopulmonary exercise testing (CPET).

**Methods:**

We analyzed 225 CHF patients who underwent standardized CPET. Using multivariate linear regression with ten-fold cross-validation, we developed prediction models for VT_1_ oxygen consumption (VO_2_) and heart rate (HR) based on readily available clinical parameters, including resting/peak exercise data, demographics, comorbidities, and medication. Model performance was evaluated using *R*^2^, root mean squared error (RMSE), mean absolute error (MAE), intraclass correlation coefficient (ICC), and Bland-Altman analysis.

**Results:**

Traditional percentage-based methods were inadequate: 80% of patients achieved VO_2_-VT_1_ at 60%–90% of peak VO_2_, and 75.6% reached HR-VT_1_ at 70%–90% of peak HR. The VO_2_-VT_1_ prediction model showed strong agreement (*R*^2^ = 0.65, RMSE = 1.42 mL/kg/min, ICC = 0.79). The HR-VT_1_ model demonstrated moderate-to-strong agreement (*R*^2^ = 0.57, RMSE = 8.4 bpm, ICC = 0.71). Bland-Altman analysis indicated good agreement for both models (Within LoA: 95.1% and 95.6%).

**Conclusion:**

CHF patients exhibit distinct exercise intensity patterns. Our validated models enable accurate, individualized estimation of VT_1_ parameters using basic clinical and exercise test data, offering a practical alternative to full CPET for personalized exercise prescription in resource-limited settings.

## Graphical Abstract

**Figure:**
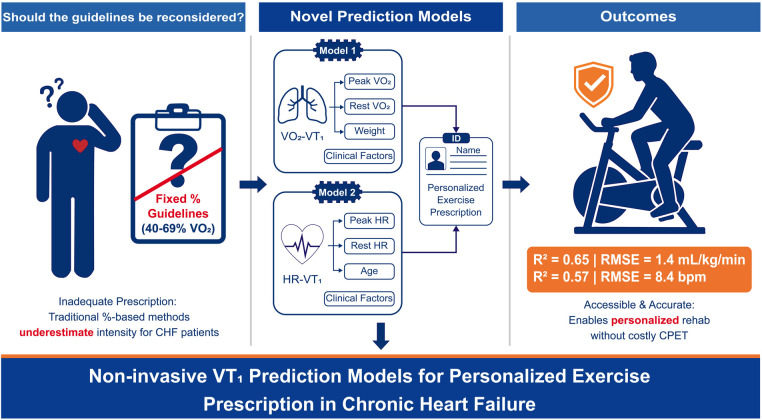

## 1 Introduction

Chronic heart failure (CHF) affects over 64 million individuals worldwide, representing a major public health challenge with profound implications for healthcare systems and patient quality of life (1, 2). The cornerstone of comprehensive CHF management lies in cardiac rehabilitation programs, where exercise training has emerged as a class I recommendation with demonstrated benefits for improving functional capacity, reducing hospitalizations, and enhancing survival outcomes (3–5). However, the therapeutic success of cardiac rehabilitation fundamentally depends on precise exercise intensity prescription, a critical factor that determines both the safety and efficacy of interventions in this vulnerable population.

Consider the clinical dilemma faced by a cardiac rehabilitation specialist treating a 65-year-old CHF patient with reduced ejection fraction. Using conventional exercise prescription guidelines that recommend 40%–69% of peak oxygen consumption (VO_2_) or 55%–75% of maximum heart rate, the prescribed intensity may systematically underestimate the patient's actual first ventilatory threshold (VT_1_), potentially limiting therapeutic benefits (4, 6, 7). Conversely, overestimation could precipitate dangerous arrhythmias or hemodynamic instability. This uncertainty highlights a fundamental gap in current practice: the extrapolation of exercise intensity recommendations from healthy populations to CHF patients without adequate validation.

The first ventilatory threshold represents a critical physiological landmark where aerobic metabolism transitions to anaerobic pathways, making it an initial optimal target for exercise prescription in cardiac rehabilitation (8). Unlike peak exercise parameters, which are heavily influenced by patient motivation, effort level, medication effects, and testing conditions, VT_1_ provides an objective, reproducible measure of exercise capacity that is less influenced by these confounding factors (9). This characteristic is particularly crucial for fragile heart failure (HF) patients who may not achieve maximal effort during cardiopulmonary exercise testing (CPET) due to fatigue, dyspnea, or musculoskeletal limitations (10). Moreover, a recent systematic review and meta-analysis demonstrated that improvement in endurance capacity during cardiac rehabilitation may be detected more accurately with the assessment of VO_2_ at VT_1_ rather than with peak VO_2_, as peak VO_2_ showed a waning effect over the long term whereas VO_2_ at VT_1_ continued to rise and was independently associated with age and left ventricular ejection fraction (11). These findings suggest that VT_1_ not only provides a more stable and reproducible endpoint for exercise prescription but also captures training-induced adaptations that may be missed by peak parameters alone, particularly in clinically vulnerable populations. However, accurate VT_1_ determination traditionally requires CPET using sophisticated equipment such as gas exchange analyzers, specialized ergometers, and trained personnel—resources that remain inaccessible to many healthcare facilities worldwide (12).

Current approaches to circumvent CPET limitations include percentage-based estimations derived from peak exercise parameters, but these methods demonstrate significant inaccuracies in CHF populations (13). Recent investigations have revealed that traditional formulas systematically misclassify exercise intensity zones, with substantial individual variations that can compromise both safety and therapeutic efficacy (14). Alternative approaches, including heart rate variability-derived thresholds and artificial intelligence-enhanced electrocardiographic analysis, show promise but require further validation in CHF-specific cohorts (15).

The physiological distinctiveness of CHF patients presents unique challenges for exercise prescription. Unlike healthy individuals, CHF patients exhibit altered hemodynamic responses, chronotropic incompetence, and medication-induced modifications of heart rate responses that fundamentally alter the relationship between exercise intensity and metabolic demand (16). Several studies have demonstrated that CHF patients can achieve superior improvements in left ventricular ejection fraction and functional capacity when exercise intensity is precisely calibrated to individual physiological thresholds rather than population-derived percentages (13, 14).

The clinical imperative for developing accessible, accurate VT_1_ prediction methods has never been more urgent (17). With the global burden of heart failure projected to increase substantially over the coming decades, healthcare systems require scalable solutions that can deliver personalized cardiac rehabilitation without dependence on expensive, specialized testing facilities (15). The development of prediction models that integrate readily available clinical parameters, such as resting and peak heart rate, basic exercise capacity measures, and patient characteristics, represents a potentially transformative approach to democratizing precision cardiac rehabilitation.

Therefore, this study aimed to develop and validate prediction models specific to patients with CHF for estimating VT_1_ parameters, utilizing comprehensive cardiopulmonary exercise testing data from a Chinese heart failure cohort. We hypothesised that (i) traditional percentage-based exercise intensity prescriptions would prove inadequate for CHF patients, and (ii) multivariate regression analysis would identify significant predictors of VT_1_ oxygen consumption and heart rate, enabling the development of clinically applicable equations. By integrating both resting and peak exercise parameters, the study sought to create validated prediction models, with performance rigorously assessed through cross-validation, intraclass correlation coefficient (ICC), and Bland-Altman analysis to ensure internal validity and clinical utility. The findings are expected to provide a more individualized and accurate framework for prescribing exercise intensity in CHF, thereby enhancing the precision and effectiveness of cardiac rehabilitation programmes.

## 2 Methods

### 2.1 Study design and population

This retrospective, single-center cohort study was conducted at Tongji Hospital Affiliated to Tongji University, analyzing chronic heart failure patients from the outpatient and cardiac rehabilitation departments between March 1, 2007, and December 31, 2018. The study protocol was approved by the Ethics Committee of Tongji Hospital Affiliated with Tongji University [LL(H)-08–04] and conducted in accordance with the 2013 revision of the Helsinki Declaration. All patients provided informed consent, and records were anonymized and de-identified before analysis.

### 2.2 Study population and selection criteria

**Figure 1:**
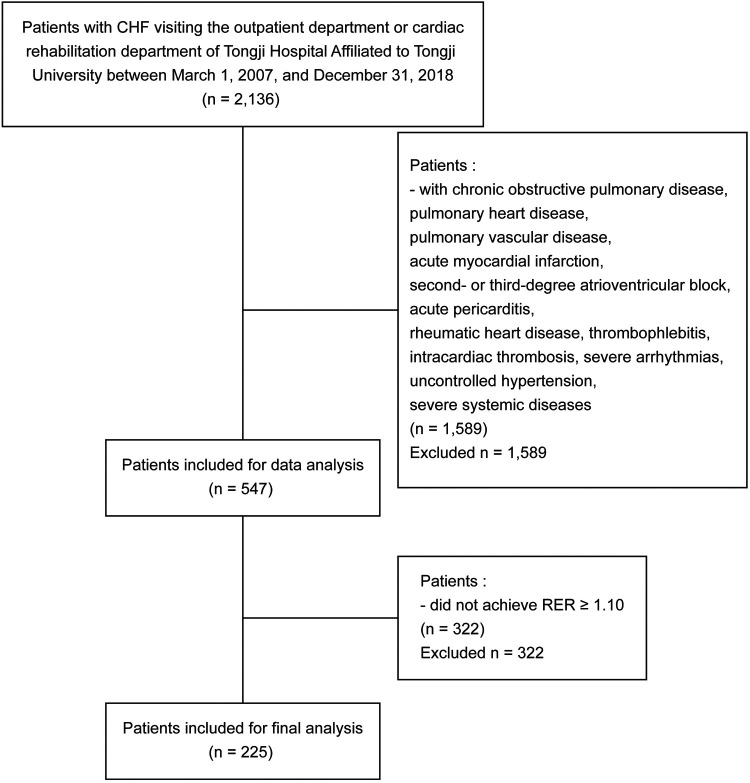
Study flowchart.

From an initial cohort of 2,136 patients, 225 CHF patients were included following rigorous selection criteria (Figure 1). Inclusion criteria comprised: (1) male and female patients diagnosed with CHF (18), (2) medical history spanning more than 3 months, (3) age ≥18 years, (4) New York Heart Association (NYHA) functional class I-III, (5) adherence to American Heart Association recommended CPET indications, and (6) successful completion of CPET with respiratory exchange ratio (RER) ≥ 1.10.

Exclusion criteria eliminated patients with chronic obstructive pulmonary disease, pulmonary heart disease, pulmonary vascular disease, acute myocardial infarction, second- or third-degree atrioventricular block, acute pericarditis, rheumatic heart disease, thrombophlebitis, intracardiac thrombosis, severe arrhythmias, uncontrolled hypertension, severe systemic diseases, motor ability limitations, RER <1.10, and failure to obtain first ventilatory threshold data from CPET.

### 2.3 Sample size calculation

Sample size calculation was performed using PASS software for effective multivariate linear regression analysis. With an unconditional model testing 35 independent variables, null hypothesis *R*^2^ = 0, alternative hypothesis *R*^2^ = 0.25 (moderate effect), significance level *α* = 0.05, and statistical power of 90%, the minimum required sample size was 128 patients (19). Accounting for potential 20% sample loss, the adjusted target was 160 patients. The final cohort of 225 patients exceeded this requirement, ensuring adequate statistical power for robust multivariate modeling.

### 2.4 Cardiopulmonary exercise testing protocol

CPET was performed using standardized equipment including the Master-Screen series lung function test system (CareFusion, Germany), Cardiosoft exercise test system (GE Medical, Germany), and Viasprint 150 electrically-braked cycle ergometer (Ergoline, Germany). The modified Ramp10 protocol consisted of three phases: 3-minute rest, 3-minute unloaded cycling, and progressive load increase from 0 J·s⁻¹ with 5 J·s⁻¹ increments every 30 s until peak exercise or termination criteria were met (20).

The first ventilatory threshold was identified using the V-slope method, detecting the breakpoint where VCO_2_ output increased sharply relative to VO_2_. To improve the reliability and confidence of threshold determination, VT_1_ identification was always confirmed by a second experienced physiologist using complementary criteria: (1) an increase in the ventilatory equivalent for oxygen (VE/VO_2_) without a simultaneous increase in the ventilatory equivalent for carbon dioxide (VE/VCO_2_), and (2) an increase in end-tidal partial pressure of oxygen (PetO_2_), as recommended in contemporary guidelines (21). All CPET procedures were conducted by qualified cardiac rehabilitation physicians and technicians who underwent standardized training to ensure consistency and data quality (20). Continuous monitoring included heart rate, oxygen uptake, carbon dioxide production, exercise load, and blood pressure throughout all phases (rest, first ventilatory threshold, and peak exercise).

### 2.5 Statistical analysis and model development

The statistical analysis was designed to develop robust prediction models for first ventilatory threshold parameters while ensuring internal validity and preventing overfitting through rigorous validation procedures.

#### 2.5.1 Variable selection and model development

A comprehensive stepwise backward elimination approach was employed to identify statistically significant predictors for VO2-VT_1_ and HR-VT_1_. The initial variable pool included 35 potential predictors encompassing demographic characteristics (age, sex, height, weight, BMI), clinical parameters (NYHA functional class, LVEF, comorbidities), medication usage (Beta-blockers, ACE inhibitors, diuretics), and cardiopulmonary exercise test measurements (resting and peak heart rate, resting and peak VO_2_, peak workload). Variables with *p*-values ≥0.05 were systematically removed from the model, with the process continuing until only statistically significant predictors (*p* < 0.05) remained.

Previous studies developing prediction equations for heart rate at ventilatory thresholds in cardiovascular disease populations have consistently identified resting heart rate and peak heart rate as independent predictors (22, 23). Therefore, our decision to include both resting and peak parameters is not only physiologically motivated but also supported by the existing literature.

The rationale for integrating both resting and peak parameters was based on physiological principles demonstrating that first ventilatory threshold represents a transitional metabolic state that cannot be adequately characterized by peak exercise parameters alone. Resting parameters reflect basal cardiovascular and metabolic function, while peak parameters indicate maximal exercise capacity. The combination provides a comprehensive physiological profile spanning the entire exercise continuum, which is particularly important in CHF patients where the relationship between resting, threshold, and peak exercise parameters differs significantly from healthy populations.

#### 2.5.2 Cross-validation and performance assessment

To ensure model robustness and prevent overfitting, a rigorous 10-fold cross-validation methodology was implemented (24). The dataset was randomly partitioned into ten equal subsets, with nine subsets used for model training and the remaining subset for validation. This process was repeated ten times, ensuring each observation served as both training and validation data. The cross-validation approach provides more reliable estimates of model performance compared to single train-test splits, particularly important given the moderate sample size of 225 patients.

#### 2.5.3 Performance metrics and model evaluation

Model performance was comprehensively evaluated using three key metrics established in medical prediction research. The coefficient of determination (*R*^2^) quantified the proportion of variance in VT_1_ parameters explained by the predictive variables. Root Mean Squared Error (RMSE) provided an absolute measure of prediction accuracy in the original units (mL/kg/min for VO_2_-VT_1_, bpm for HR-VT_1_), while Mean Absolute Error (MAE) offered a more interpretable metric representing the average magnitude of prediction errors.

#### 2.5.4 Consistency and agreement assessment using Intraclass Correlation Coefficient (ICC)

To evaluate the consistency between predicted and measured VT_1_ values, we calculated the intraclass correlation coefficient (ICC) using a two-way mixed-effects model for absolute agreement [ICC(A,1)] (25). This statistical measure assesses the reliability of measurements by comparing the variability within paired observations (predicted vs. measured) to the total variability across all observations. ICC values range from 0 to 1, with higher values indicating greater consistency. Following established benchmarks (26), we interpreted ICC values as follows: <0.5 (poor), 0.5–0.75 (moderate), 0.75–0.9 (good), and >0.9 (excellent) reliability. The ICC analysis provides a standardized metric for the agreement between our model-predicted thresholds and the CPET-derived gold-standard measurements.

#### 2.5.5 Bland-Altman analysis for assessing measurement agreement

We further employed Bland-Altman analysis to quantify the agreement between predicted and observed VT_1_ values and to identify any systematic bias (27). For each VT_1_ parameter (VO_2_ and HR), the difference between the predicted and measured value was plotted against their mean. The mean difference (bias) and its 95% confidence interval were calculated to estimate systematic over- or underestimation. The 95% limits of agreement (LoA) were defined as bias ± 1.96 × standard deviation of the differences, representing the range within which 95% of the differences between the two methods are expected to lie. The width of the LoA provides a clinically relevant measure of prediction precision, while the distribution of points relative to the zero-bias line visually reveals patterns of heteroscedasticity or proportional error.

#### 2.5.6 Mathematical formulation and model assumptions

The prediction models were formulated as multivariate linear regression equations of the form:

$$Y=\beta_{0}+\beta_{1}X_{1}+\beta_{2}X_{2}+\ldots +\beta_{n}X_{n}+\varepsilon$$

where Y represents the dependent variable (VO2-VT_1_ or HR-VT_1_), *β*₀ is the intercept, *β*ᵢ are regression coefficients for predictor variables Xᵢ, and *ε* represents the error term. Model assumptions including linearity, independence of residuals, homoscedasticity, and normality of residuals were systematically tested using standard diagnostic procedures.

#### 2.5.7 Missing data and quality control

Missing data handling followed established protocols for clinical research, with complete case analysis employed given the relatively low proportion of missing values (<5%). Sensitivity analyses were conducted to assess the impact of missing data on model performance. All statistical analyses were performed using IBM SPSS Statistics 22.0, R language (version 4.2.1), and Python Environments (v1.16.0), with significance levels set at *α* = 0.05. The analytical approach ensured that the developed models would provide clinically meaningful and statistically robust predictions for VT_1_ parameters in CHF patients while maintaining transparency and reproducibility in the modeling process.

## 3 Results

### 3.1 Patient characteristics

The study cohort comprised 225 Chinese CHF patients with a median age of 62 years, predominantly male (81.8%), and a median left ventricular ejection fraction of 45% (Table 1). The population exhibited typical comorbidities of CHF, including coronary artery disease (67.1%), hypertension (68.4%), and diabetes mellitus (27.6%). These characteristics are representative of the East Asian CHF population and align with prior epidemiological reports (28).

**Table 1:** Baseline demographic and clinical characteristics of CHF patients.

	Population (*n* = 225)	Male (*n* = 184)	Female (*n* = 41)	*χ*^2^/*Z*/t	*P*
Height, cm	168 ± 7	170 ± 5	158 ± 6	14.217	<0.001
Weight, kg	71 ± 12	73 ± 11	63.0 ± 12.8	5.091	<0.001
Waistline, cm	91.9 ± 11.3	92.3 ± 11.0	90.3 ± 12.8	1.035	0.302
BMI, kg/m^2^	25.0 ± 3.4	25.0 ± 3.2	25.1 ± 4.1	−0.170	0.865
Waist-to-high ratio	0.55 ± 0.06	0.54 ± 0.06	0.57 ± 0.73	−2.610	0.009
LVEF	0.44 ± 0.10	0.44 ± 0.12	0.46 ± 0.12	−1.279	0.202
Cigarette, *n* (%)	140 (62.2%)	132 (71.7%)	8 (19.5%)	38.907	<0.001
Alcohol, *n* (%)	34 (15.1%)	34 (18.5%)	–	8.925	0.003
NYHA class, *n* (%)				1.639	0.441
Ⅰ	25 (11.1%)	22 (11.9%)	3 (7.3%)		
Ⅱ	152 (67.6%)	121 (65.7%)	31 (75.6%)		
Ⅲ	48 (21.3%)	41 (22.2%)	7 (17.0%)		
Ⅳ	–	–	–		
Diagnosis, *n* (%)					
ICM	151 (67.1%)	127 (69.0%)	24 (58.5%)	2.374	0.305
DCM	58 (25.8%)	46 (25.0%)	12 (29.2%)	2.436	0.119
CAD	151 (67.1%)	127 (69.0%)	24 (58.5%)	1.670	0.196
PCI	103 (45.8%)	89 (48.3%)	14 (34.1%)	2.733	0.098
MI	95 (42.2%)	82 (44.5%)	13 (31.7%)	2.272	0.132
AF	29 (12.9%)	21 (11.4%)	8 (19.5%)	1.959	0.162
Complication, *n* (%)					
Type 2 diabetes	62 (27.6%)	50 (27.1%)	12 (29.2%)	0.074	0.786
Hypertension	154 (68.4%)	125 (67.9%)	29 (70.7%)	0.121	0.727
Hyperlipidemia	161 (71.6%)	136 (73.9%)	25 (60.9%)	2.757	0.097
Drugs, *n* (%)					
Digoxin	42 (18.7%)	35 (19.0%)	7 (17.0%)	0.084	0.772
Diuretics	79 (35.1%)	58 (31.5%)	21 (51.2%)	5.710	0.017
Nitrates	56 (24.9%)	43 (23.3%)	13 (31.7%)	1.247	0.264
ACEI/ARB	175 (77.8%)	145 (78.8%)	30 (73.1%)	0.616	0.433
Beta blockers	194 (86.2%)	161 (87.5%)	33 (80.4%)	1.388	0.240
Antiplatelets	166 (73.8%)	139 (75.5%)	27 (65.8%)	1.627	0.202
Anticoagulants	27 (12.0%)	16 (8.6%)	11 (26.8%)	10.441	0.001
Hypolipidemic agents	161(71.6%)	136(73.9%)	25(60.9%)	2.757	0.097

BMI, Body Mass Index; LVEF, Left Ventricular Ejection Fraction; NYHA, New York Heart Association; ICM, Ischemic Cardiomyopathy; DCM, Dilated Cardiomyopathy; CAD, Coronary Artery Disease; PCI, Percutaneous Coronary Intervention; MI, Myocardial Infarction; AF, Atrial Fibrillation; ACEI, Angiotensin-Converting Enzyme Inhibitor; ARB, Angiotensin Receptor Blocker.

### 3.2 Exercise parameters and traditional method limitations

The comprehensive cardiopulmonary exercise testing results revealed significant differences in exercise capacity parameters between male and female CHF patients, with important implications for exercise prescription methodologies. As presented in Table 2, male patients demonstrated superior exercise performance across multiple parameters while maintaining similar cardiovascular responses during exercise testing.

**Table 2:** Results of cardiopulmonary exercise test for male and female CHF patients.

	Population (*n* = 225)	Male (*n* = 184)	Female (*n* = 41)	χ^2^/*Z*/t	*P*
HRR, bmp	46 ± 16	45 ± 16	48 ± 18	−0.941	0.348
VE/VCO_2_ slope	36.6 ± 8.8	35.8 ± 8.2	39.9 ± 10.3	−2.740	0.007
Rest parameters					
rest HR, bpm	72 ± 6	72 ± 7	72 ± 5	0.531	0.596
rest SBP, mmHg	116 ± 12	116 ± 12	117 ± 15	−0.486	0.627
rest DBP, mmHg	73 ± 6	73 ± 6	72 ± 5	1.609	0.109
rest VO_2_, mL/kg/min	4.5 ± 1.3	4.5 ± 1.4	4.1 ± 1.1	4,274	0.183
First ventilatory threshold parameters					
VT_1_ HR, bpm	95 ± 13	94 ± 12	98 ± 15	−1.661	0.098
VT_1_ SBP, mmHg	137.1 ± 14.3	136.7 ± 14.0	139.2 ± 15.3	−1.080	0.280
VT_1_ DBP, mmHg	77.8 ± 6.6	77.8 ± 8.7	78.0 ± 2.3	−0.492	0.623
VT_1_ Load, watt	34.3 ± 11.9	36.1 ± 10.7	25.9 ± 9.6	3.184	0.002
VT_1_ VO_2_, mL/kg/min	11.0 ± 2.4	11.2 ± 2.4	10.1 ± 2.2	2.552	0.011
Peak parameters					
peak HR, bpm	118 ± 18	118 ± 16	120 ± 19	0.531	0.596
peak SBP, mmHg	151 ± 26	151 ± 26	152 ± 22	−0.273	0.813
peak DBP, mmHg	81 ± 14	81 ± 15	80 ± 12	0.656	0.512
peak Load, watt	69.6 ± 24.4	73.0 ± 24.2	54.2 ± 19.1	4.650	<0.001
peak VO_2_, mL/kg/min	15.2 ± 3.7	15.6 ± 3.8	13.3 ± 2.9	5,176.5	2e-04
VT_1_ as Percentage of Peak					
HR-VT_1_ as Percentage of Peak HR, %	80.8 ± 8.6	80.5 ± 8.7	82.1 ± 8	−1.159	0.251
%HRR-VT_1_, %	49.4 ± 19.5	48.5 ± 19.3	53.5 ± 20	−1.450	0.153

HRR, Heart Rate Reserve; VE, Minute Ventilation; VCO_2_, Carbon Dioxide Excretion; HR, Heart Rate; SBP, Systolic Blood Pressure; DBP, Diastolic Blood Pressure; VO_2_, Oxygen Consumption; VT_1_, First Ventilatory Threshold.

Male patients exhibited significantly higher oxygen consumption values across all exercise phases. Resting VO_2_ was substantially higher in males (4.5 ± 1.4 mL/kg/min vs. 4.1 ± 1.1 mL/kg/min, *p* = 0.183), which translated to superior performance at the first ventilatory threshold (11.2 ± 2.4 mL/kg/min vs. 10.1 ± 2.2 mL/kg/min, *p* = 0.011) and peak exercise (15.6 ± 3.8 mL/kg/min vs. 13.3 ± 2.9 mL/kg/min, *p* < 0.001). Correspondingly, male patients achieved higher workloads at both first ventilatory threshold (36.1 ± 10.7 watts vs. 25.9 ± 9.6 watts, *p* < 0.01) and peak exercise (73.0 ± 24.2 watts vs. 54.2 ± 19.1 watts, *p* < 0.001). Notably, despite these performance differences, heart rate responses and blood pressure parameters showed remarkable consistency between genders across all exercise phases.

### 3.3 Traditional exercise prescription methods prove inadequate for CHF patients

**Figure 2:**
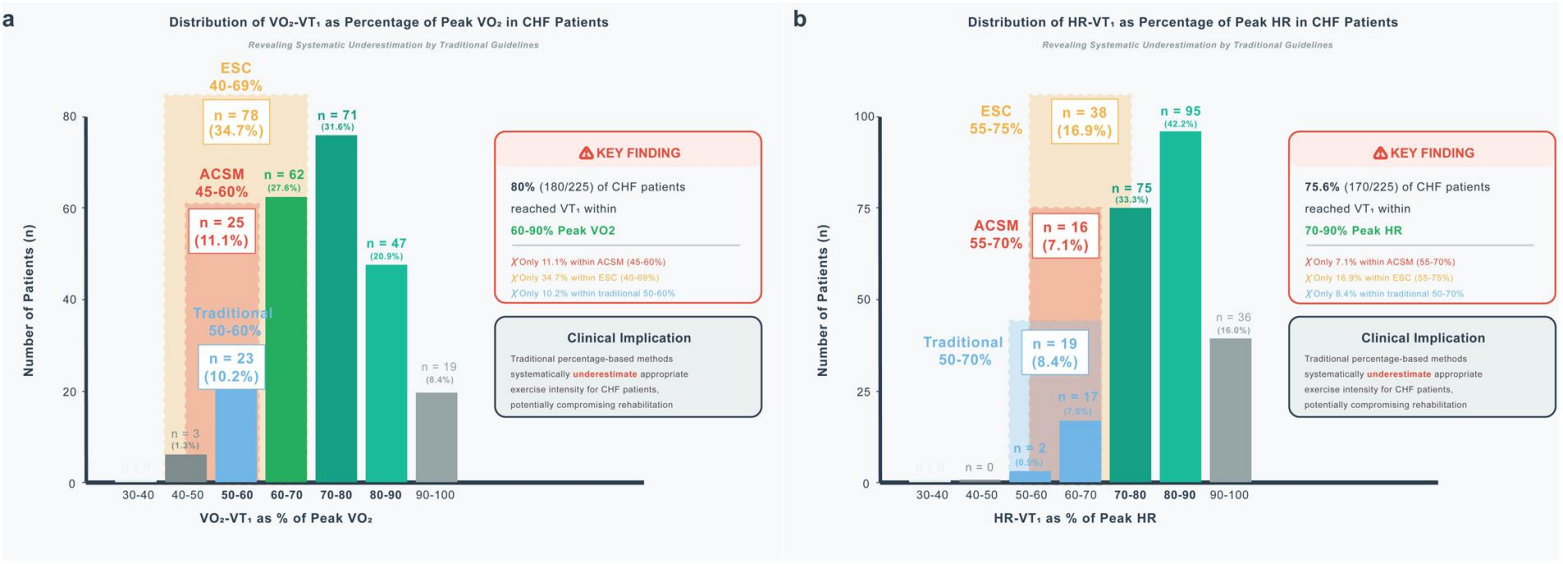
Distribution of VT_1_ value as percentage of peak value in CHF patients. **(a)** Distribution of VO_2_-VT_1_ as Percentage of Peak VO_2_ in CHF Patients. Among all patients with chronic heart failure, a significant proportion (80%, a total of 180 cases) reached their first ventilatory threshold, which accounted for 60% to 90% of their maximum oxygen uptake. Traditional percentage-based methods systematically underestimate appropriate exercise intensity for CHF patients, potentially compromising rehabilitation. **(b)** Distribution of HR-VT_1_ as Percentage of Peak HR in CHF Patients. The majority of CHF patients (75.6%, *n* = 170) reached their first ventilatory threshold at 70–90% of peak heart rate. Traditional percentage-based methods systematically underestimate appropriate exercise intensity for CHF patients, potentially compromising rehabilitation.

Analysis of VT_1_ parameter distributions revealed profound discrepancies between actual physiological thresholds and guideline-recommended intensity ranges (Table 3). The majority of CHF patients (80%, *n* = 180/225) achieved their first ventilatory threshold between 60% and 90% of peak VO_2_ (Figure 2a), dramatically exceeding traditional exercise prescription recommendations. Only 11.1% (*n* = 25) of patients fell within the American College of Sports Medicine's recommended 45%–60% peak VO_2_ range, while 34.7% (*n* = 78) aligned with the European Society of Cardiology's broader 40%–69% range (4, 29). The commonly cited 50%–60% peak VO_2_ equivalent captured merely 10.2% (*n* = 23) of the study population.

**Table 3:** Comparison of guideline recommendations vs. Actual VT_1_ parameters in CHF patients.

Guideline/Method	Recommended range	Actual AT distribution	Agreement rate	Systematic bias
VO_2_-VT_1_ prescription				
ACSM	45%–60% Peak VO_2_	60%–90% (80%)	11.1%	−22.5% underestimation
ESC	40%–69% Peak VO_2_		34.7%	−20.5% underestimation
Traditional 50%–60%	50%–60% Peak VO_2_		10.2%	−20.0% underestimation
HR-VT_1_ Prescription				
ACSM	55%–70% Peak HR	70%–90% (75.6%)	7.1%	−16.9% underestimation
ESC	55%–75% Peak HR		16.9%	−14.4% underestimation
Traditional 60%–70%	60%–70% Peak HR		8.4%	−14.4% underestimation

Agreement rate = percentage of patients whose actual VT_1_ falls within recommended range. Systematic bias calculated as mean difference between recommended midpoint and actual VT_1_ parameter.

VO_2_-VT_1_, Oxygen Consumption at first ventilatory threshold; ACSM, American College of Sports Medicine; ESC, European Society of Cardiology; VO_2_, Oxygen Consumption; HR-VT_1_, Heart Rate at first ventilatory threshold; HR, Heart Rate.

Similarly striking patterns emerged for heart rate parameters (Figure 2b). The majority of CHF patients (75.6%, *n* = 170) reached their first ventilatory threshold at 70%–90% of peak heart rate, substantially higher than traditional recommendations. Only 7.1% (*n* = 16) of patients fell within the American College of Sports Medicine's suggested 55%–70% peak heart rate range, 16.9% (*n* = 38) within the European Society of Cardiology's 55%–75% range, and 8.4% (*n* = 19) within the generally accepted 50%–70% range.

### 3.4 Prediction model performance and validation

Through comprehensive multivariate linear regression analysis incorporating 10-fold cross-validation, two robust prediction models were successfully developed for estimating first ventilatory threshold parameters in chronic heart failure patients.

#### 3.4.1 VO_2_-VT_1_ prediction model

**Figure 3:**
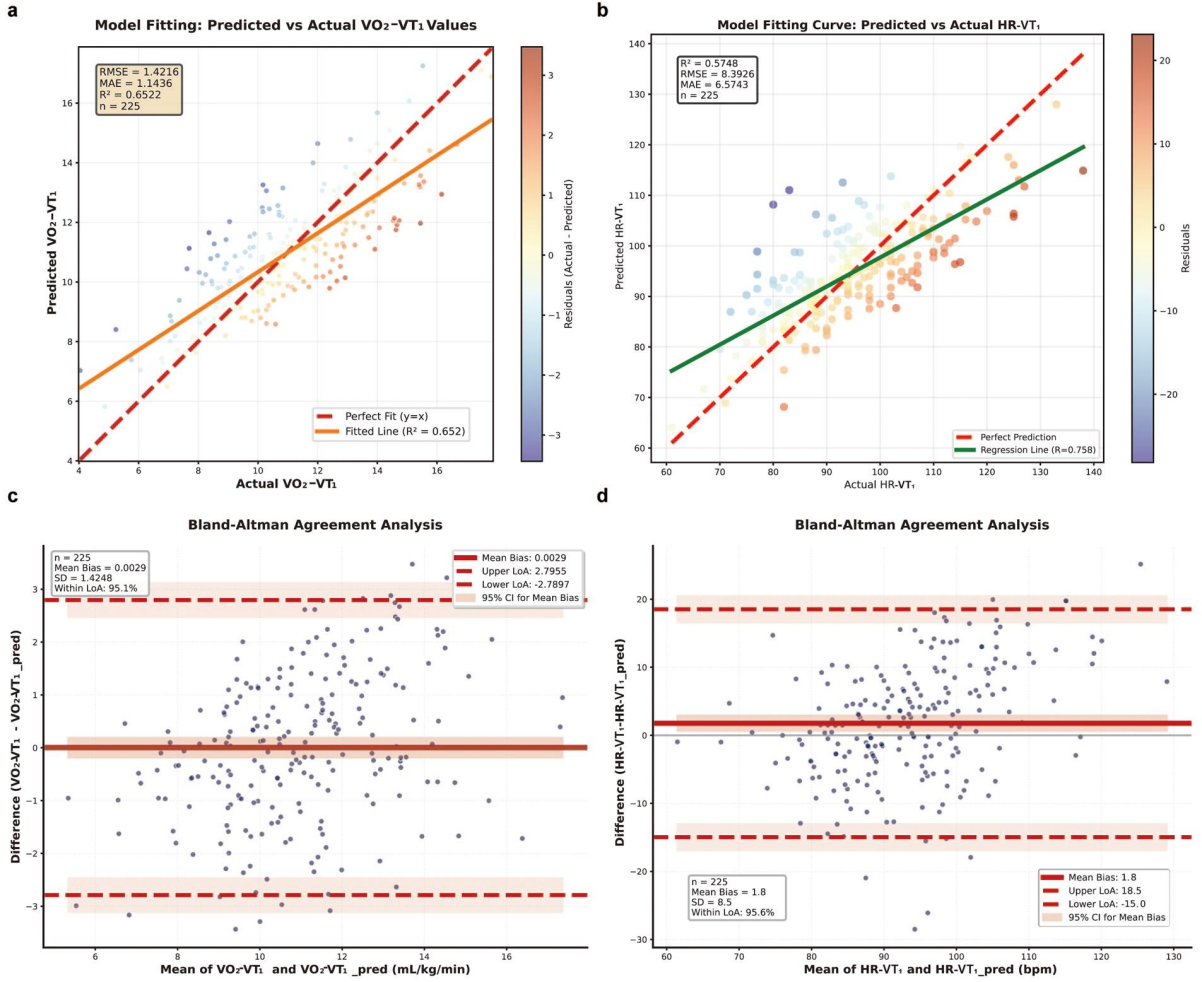
Prediction model in CHF patients. **(a)** VO_2_-VT_1_ Prediction Model in CHF Patients. **(b)** HR-VT_1_ Prediction Model in CHF Patients. **(c)** Bland-Altman plots for the comparison between predicted vs. actual VO_2_-VT_1_. **(d)** Bland-Altman plots for the comparison between predicted vs. actual HR-VT_1_.

The VO_2_-VT_1_ prediction model demonstrated exceptional performance with an adjusted *R*^2^ of 0.6522, indicating that the model explains approximately 65.22% of the variance in first ventilatory threshold oxygen consumption (Figure 3a). The complete mathematical formula is:

$$\begin{array}{rl} VO_{2}-VT_{1}= & 0.501\times \mathrm{peak}\;VO_{2}+0.159\times \mathrm{rest}\;VO_{2}-0.432\\ & \;\times [\mathrm{Smoking}\;\mathrm{history}]-0.520\\ & \;\times [\mathrm{Coronary}\;\mathrm{artery}\;\mathrm{disease}]\\ & \;-\;0.790\times [\mathrm{Atrial}\;\mathrm{fibrillation}]+0.346\\ & \;\times [\mathrm{Hypertension}]\;+0.523\times [\mathrm{Beta}\;\mathrm{blockers}]\\ & \;+2.660 \end{array}$$

The model achieved remarkable accuracy with a root mean squared error (RMSE) of 1.42 mL/kg/min and mean absolute error (MAE) of 1.14 mL/kg/min. Cross-validation analysis confirmed the model's robustness and internal validity, with consistent performance across all ten validation folds.

Variable contribution analysis revealed that peak VO_2_ was the strongest predictor (coefficient: 0.501, *p* < 0.001), followed by resting VO_2_ (coefficient: 0.159, *p* = 0.033), demonstrating the critical importance of integrating both baseline and maximal exercise parameters. Among clinical comorbidities, atrial fibrillation exerted the strongest negative effect (coefficient: −0.790, *p* = 0.007), followed by coronary heart disease (coefficient: −0.520, *p* = 0.019) and smoking history (coefficient: −0.432, *p* = 0.037). Hypertension and beta blockers use showed weak positive associations (*p* > 0.05).

#### 3.4.2 HR-VT_1_ prediction model

The HR-VT_1_ prediction model achieved satisfactory performance with an adjusted *R*^2^ of 0.57, explaining 57% of the variance in first ventilatory threshold heart rate (Figure 3b). The mathematical formulation is:

$$\begin{array}{rl} \mathrm{HR}-VT_{1}= & 0.555\times \mathrm{rest}\;\mathrm{HR}+0.485\times \mathrm{peak}\;\mathrm{HR}+0.122\\ & \;\times \mathrm{Age}-3.383\times [\mathrm{Coronary}\;\mathrm{artery}\;\mathrm{disease}]\\ & \;-9.547 \end{array}$$

Performance metrics showed an RMSE of 8.4 bpm and MAE of 6.6 bpm, indicating clinically acceptable prediction accuracy. The model demonstrated balanced contributions from both peak HR (coefficient: 0.485) and resting HR (coefficient: 0.555), reinforcing the necessity of incorporating baseline cardiovascular status.

Age emerged as a significant positive predictor (coefficient: 0.122), while coronary heart disease showed a negative association (coefficient: −3.383).

#### 3.4.3 Model validation and consistency analysis

Cross-validation results demonstrated good model stability, with minimal variation in performance metrics across validation folds. The VO_2_-VT_1_model showed superior performance compared to traditional single-parameter approaches, with the integration of both resting and peak parameters providing significantly better predictive accuracy than models using peak parameters alone.

Calibration analysis revealed moderate-to-good agreement between predicted and observed values, with the model fitting curves closely approximating the ideal prediction line. The scattered distribution of measured values around the fitting curve indicated good generalization ability with a small mean bias (1.8 bpm for HR-VT1 and 0.0029 mL/kg/min for VO_2_-VT1), which was statistically significant but clinically small.

Calibration and predictive accuracy assessment confirmed the models' ability to accurately stratify patients across the full range of AT values. The VO_2_-VT_1_model demonstrated particularly strong discriminative power in the clinically relevant range of approximately 8–12 mL/kg/min, while the HR-VT_1_ model showed consistent performance across heart rate ranges of approximately 80–120 bpm.

This study conducted a comprehensive consistency analysis of VO_2_-VT_1_ (actual measured value) and VO_2_-VT_1__pred (predicted value) for 225 patients (Table 4). Through descriptive statistics, correlation analysis, Bland-Altman consistency analysis, ICC, and paired statistical tests, the consistency between the two measurement methods was evaluated. The results showed a strong correlation between the two variables (r = 0.8076), but there was a statistically significant systematic bias, with the predicted value averaging underestimating the actual value by 0.0029 mL/kg/min. Bland-Altman analysis for the comparison between predicted vs. actual VO_2_-VT_1_ is shown in Figure 3c. Besides, the ICC value of VO2-VT_1_ prediction model is 0.7902, indicating a good consistency between VO_2_-VT_1_ and VO_2_-VT_1__pred.

**Table 4:** Consistency analysis between predicted and actual value.

Metric	Predicted vs. actual VO_2_-VT_1_		Predicted vs. actual HR-VT_1_	
	Value	*P* value	Value	*P* value
Pearson Correlation	0.8076	<0.001	0.7492	<0.001
Spearman Correlation	0.7833	<0.001	0.7185	<0.001
ICC	0.7902	N/A	0.7122	N/A
RMSE	1.42	N/A	8.7	N/A
MAE	1.14	N/A	6.8	N/A
Mean Bias	0.0029	N/A	1.8	N/A
Standard Deviation of Differences	1.4248	N/A	8.5	N/A
Upper 95% LoA	2.7955	N/A	18.5	N/A
Lower 95% LoA	−2.7897	N/A	−15.0	N/A
Within LoA	95.1%	N/A	95.6%	N/A

ICC, Intraclass Correlation; RMSE, Root Mean Square Error; MAE, Mean Absolute Error; LoA, Limits of Agreement.

Similarly, this report analyzed the consistency between the HR-VT_1_ (actual value) and HR-VT_1__pred (predicted value) variables (Table 4). Through various statistical methods including correlation analysis, ICC, and Bland-Altman analysis, the accuracy and reliability of the prediction model were evaluated. The analysis results showed a good positive correlation between the two variables (r = 0.749), but the predicted values were slightly lower than the actual values, with an average deviation of 1.77. Besides, the ICC value of HR-VT_1_ prediction model is 0.7122, indicating a good consistency between HR-VT_1_ and HR-VT_1__pred. Bland-Altman analysis for the comparison between predicted vs. actual HR-VT_1_ is shown in Figure 3d. A mean bias of 1.8 bpm was found, while limits of agreement ranged between −15.0 bpm and 18.5 bpm.

## 4 Discussion

Our findings unequivocally demonstrate that conventional percentage-based methods for prescribing exercise intensity are fundamentally mismatched to the physiology of patients with CHF. The vast majority of our cohort (80%) achieved their VT_1_ at 60%–90% of peak VO_2_, starkly contrasting with guideline-recommended ranges of 40%–69% (4, 29). This systematic underestimation, averaging nearly 20%, corroborates and extends recent critiques of existing guidelines. While studies in general cardiovascular populations have highlighted inconsistencies (14, 30), our data provide the first robust, quantitative evidence specifically within a Chinese CHF cohort, revealing a more pronounced discrepancy than previously suggested. These findings reveal a fundamental inadequacy in current percentage-based exercise prescription methods when applied to CHF populations. The magnitude of prediction errors is clinically substantial, with traditional approaches potentially underestimating appropriate exercise intensity for the vast majority of CHF patients. This systematic underestimation could result in suboptimal rehabilitation outcomes, as patients may receive exercise prescriptions below their therapeutic threshold, limiting the cardiovascular benefits of cardiac rehabilitation programs.

This study represents a significant advance beyond prior research by transitioning from merely identifying the problem to delivering a validated, practical solution. Earlier work effectively critiqued the misalignment between guideline-based percentages and physiological thresholds but stopped short of offering alternative, accessible prediction tools (13, 30). It is important to acknowledge that previous studies, notably by Milani et al., have developed and externally validated prediction equations for heart rate at ventilatory thresholds in mixed populations with cardiometabolic disease (22). While Milani et al. derived equations from a mixed cohort of coronary artery disease, heart failure, and other diagnoses, our study is the first to develop VT_1_ prediction models exclusively in a CHF population, and the first in an Asian (Chinese) cohort. Notably, Milani et al. also reported that resting heart rate and peak heart rate were the strongest independent predictors of heart rate at VT_1_ in their mixed cohort, which aligns closely with our findings and further justifies the integration of both parameters. Moreover, our models provide both VO_2_-VT_1_ and HR-VT_1_ equations using a parsimonious set of variables that are readily available in routine clinical practice. Thus, we bridge this critical gap by developing the first multivariate linear regression models specifically for VT_1_ prediction in a CHF population, moving the field from critique to implementation.

A key aspect of our methodological approach is the synergistic integration of both resting and peak exercise parameters. While previous studies have incorporated similar parameters (e.g., resting and peak HR), our study applies this approach specifically to a CHF-only population and provides prediction equations for both VO_2_ and HR at VT_1_, which has not been previously reported. The physiological rationale is compelling: VT_1_ is a transitional metabolic state (31). Relying solely on peak capacity (e.g., peak VO_2_) ignores the crucial influence of baseline cardiometabolic function, which is particularly deranged in CHF (32). Our models mathematically encode this relationship, explaining a substantially higher proportion of variance (*R*^2^ = 0.65) than models using peak parameters alone. This integrative approach offers a more holistic representation of the patient's exercise physiology continuum.

A key innovation is the balance our models strike between precision and accessibility. While the gold-standard CPET remains indispensable for comprehensive assessment, its cost and complexity limit widespread use (33). Alternative non-CPET methods, such as heart rate variability-derived thresholds (e.g., DFA a1), show promise but require validation in larger CHF cohorts and specialized analysis (15). Our models, in contrast, utilize parameters (resting and peak HR, workload, basic demographics, and clinical history) that can be obtained through a standard symptom-limited exercise test without mandatory gas analysis. This makes personalized prescription feasible in community hospitals and telerehabilitation settings where full CPET is unavailable, directly addressing a major healthcare accessibility barrier.

The clinical value of VT_1_-based exercise prescription extends beyond the statistical advantages demonstrated in our prediction models, particularly when considering the most fragile HF patients—those with advanced NYHA class, low functional capacity, or multiple comorbidities. For these patients, achieving maximal effort during CPET is often challenging due to fatigue, dyspnea, or chronotropic incompetence, rendering peak parameters less reliable as a basis for exercise prescription. In contrast, VT_1_ is a submaximal threshold that can be identified even when patients terminate exercise before reaching true maximal capacity, making it a more accessible and patient-centered target for rehabilitation (23).

Furthermore, emerging evidence supports the superiority of VT_1_-based prescription over peak-based methods for monitoring training-induced adaptations. A systematic review and meta-analysis by Christou et al. found that improvement in endurance capacity during cardiac rehabilitation may be detected more accurately with VO_2_ at VT_1_ than with peak VO_2_ (11). Importantly, while peak VO_2_ showed a negative association with training duration (coefficient = −0.061, *p* = 0.027), suggesting a waning effect of long-term exercise training.VO_2_ at VT_1_ was independently predicted by both age (coefficient = −0.027, *p* = 0.064) and LVEF (coefficient = 2.859, *p* = 0.037), highlighting its sensitivity to clinically meaningful physiological factors. These findings align with observations in athletic populations, where VO_2_ at VT_1_ continues to rise even when peak VO_2_ has reached a plateau, suggesting that VT_1_ captures ongoing submaximal adaptations that may be masked by peak parameters.

From a clinical safety perspective, prescribing exercise at VT_1_ intensity avoids the risks associated with high-intensity exercise (e.g., arrhythmias, hemodynamic instability) while ensuring that patients train at a sufficient load to elicit meaningful cardiovascular benefits. This balance is particularly critical in fragile HF patients, who may have narrower safety margins. By enabling personalized prescription based on the patient's own physiological transition point rather than population-derived percentages, VT_1_-guided exercise reduces the likelihood of both undertraining (subtherapeutic effects) and overtraining (adverse events). Indeed, a recent position statement from the European Association of Preventive Cardiology identified prescription based on ventilatory thresholds as the preferable methodology in cardiac rehabilitation (21).

The improved performance of our multi-parameter models compared to traditional percentage-based approaches was evident in their ability to account for individual patient characteristics. The integration of resting parameters provided crucial baseline metabolic and cardiovascular information, while peak parameters captured maximal exercise capacity. Clinical variables including comorbidities and medications added important prognostic context, resulting in personalized predictions that reflect the complex pathophysiology of chronic heart failure. Specifically, the incorporation of clinical variables (e.g., atrial fibrillation, coronary artery disease, smoking history) represents another layer of innovation, enabling true personalization. These factors, with their significant negative coefficients in our VO_2_-VT_1_ model, quantitatively capture the impact of comorbidities on exercise tolerance, which is a nuance completely absent in one-size-fits-all percentage formulas. This allows clinicians to adjust exercise prescriptions not just based on capacity, but on the individual's specific clinical profile, enhancing both safety and potential efficacy. The models' robust performance metrics, combined with their practical applicability using readily available clinical data, represent a significant advancement in non-invasive VT_1_ parameter estimation for CHF patients, offering a viable alternative to expensive and technically demanding cardiopulmonary exercise testing. We recognize that guideline-directed %HRR is another valuable approach for exercise prescription; however, a formal comparison with %HRR was not the primary focus of this study. Descriptive %HRR data at VT1 are provided in Table 2 and Supplementary Table 1^1^.

Beyond the comparisons with guideline-recommended percentage-of-peak methods, the percentage of heart rate reserve (%HRR) represents another widely advocated approach for individualizing exercise intensity (34). %HRR incorporates resting heart rate and has been shown to provide a closer approximation to VT_1_ than %peak HR alone, particularly in patients with chronotropic incompetence or those on *β*-blockers (34). However, the application of %HRR in CHF populations has important limitations. Its equivalence with %VO_2_R has not been rigorously validated in CHF, and the recommended %HRR range (typically 40%–59%) is derived largely from healthy cohorts and may not correspond to VT_1_ in CHF patients (35–37).

Given the above considerations—and because the primary aim of our study was to develop practical prediction models for VT_1_ parameters using readily available clinical data, rather than to assess the accuracy of existing %HRR recommendations—we chose not to include a formal %HRR comparison in the present investigation. Our decision was based on two reasons: (1) our focus was on guideline-referenced methods expressed as percentages of peak VO_2_ or peak HR, which are the direct targets of current clinical practice; (2) adding a %HRR comparison would have expanded the scope of the study without directly contributing to the development of our VT_1_ prediction equations. We do, however, agree that %HRR is a valuable method that deserves further investigation. Future studies should compare %HRR, %peak HR, and our equations head-to-head in larger CHF cohorts.

We acknowledge limitations inherent in our single-center, retrospective design, which may affect generalizability to other ethnicities or healthcare systems. External validation in diverse populations is a necessary next step. In addition, our cohort was predominantly male (81.8%), which reflects the epidemiology of CHF in our population but limits the generalizability of our findings to female patients. Sex-related differences in cardiovascular and chronotropic responses have been well documented, and future studies should validate our models in a larger and more balanced female cohort. Additionally, the stepwise variable selection was performed using the entire dataset before cross-validation, which may introduce a degree of optimism in the performance estimates; future external validation should employ a nested cross-validation approach to fully address this limitation. Specifically, future validation should be performed in real-world clinical settings, including heart failure patients who are unable to achieve a respiratory exchange ratio (RER) ≥ 1.10 during cardiopulmonary exercise testing due to fatigue, dyspnea, or chronotropic incompetence. Such validation would better reflect routine clinical practice and further establish the external applicability of our prediction models. Furthermore, while our models improve accessibility, they still require a measured peak workload, which may not be available for all severely debilitated patients. Future research should explore integration with wearable device data and machine learning algorithms to further simplify input requirements and enhance predictive accuracy for broader application.

## 5 Conclusion

This study demonstrates that traditional percentage-based exercise intensity prescriptions are inadequate for CHF patients, with the majority achieving VT_1_ at significantly higher percentages of peak VO_2_ and HR than guideline-recommended ranges. We have developed and validated two prediction models for VO2-VT_1_ and HR-VT_1_ that utilize readily available clinical and exercise test parameters. These models offer a practical, non-invasive alternative to full CPET, enabling personalized exercise prescription in resource-limited settings. Future research should focus on external validation in diverse populations and integration into digital health platforms to further enhance accessibility and usability.

## Data Availability

The data presented in this study are available on request from the corresponding author. Requests to access these datasets should be directed to Yuqin Shen MD, PhD sy_1963@126.com.
