# Supplementary material for: Non-invasive prediction of the first ventilatory threshold in Chinese patients with chronic heart failure for personalized exercise prescription
Source: Front Cardiovasc Med. 2026 Jul 6;13:1805304. doi: 10.3389/fcvm.2026.1805304 (PMC13381753; doi:10.3389/fcvm.2026.1805304)
Supplement: Supplementary file 1 [file Datasheet1.docx]

Supplementary Material

# Supplementary Figures and Tables

## Supplementary Figures

**
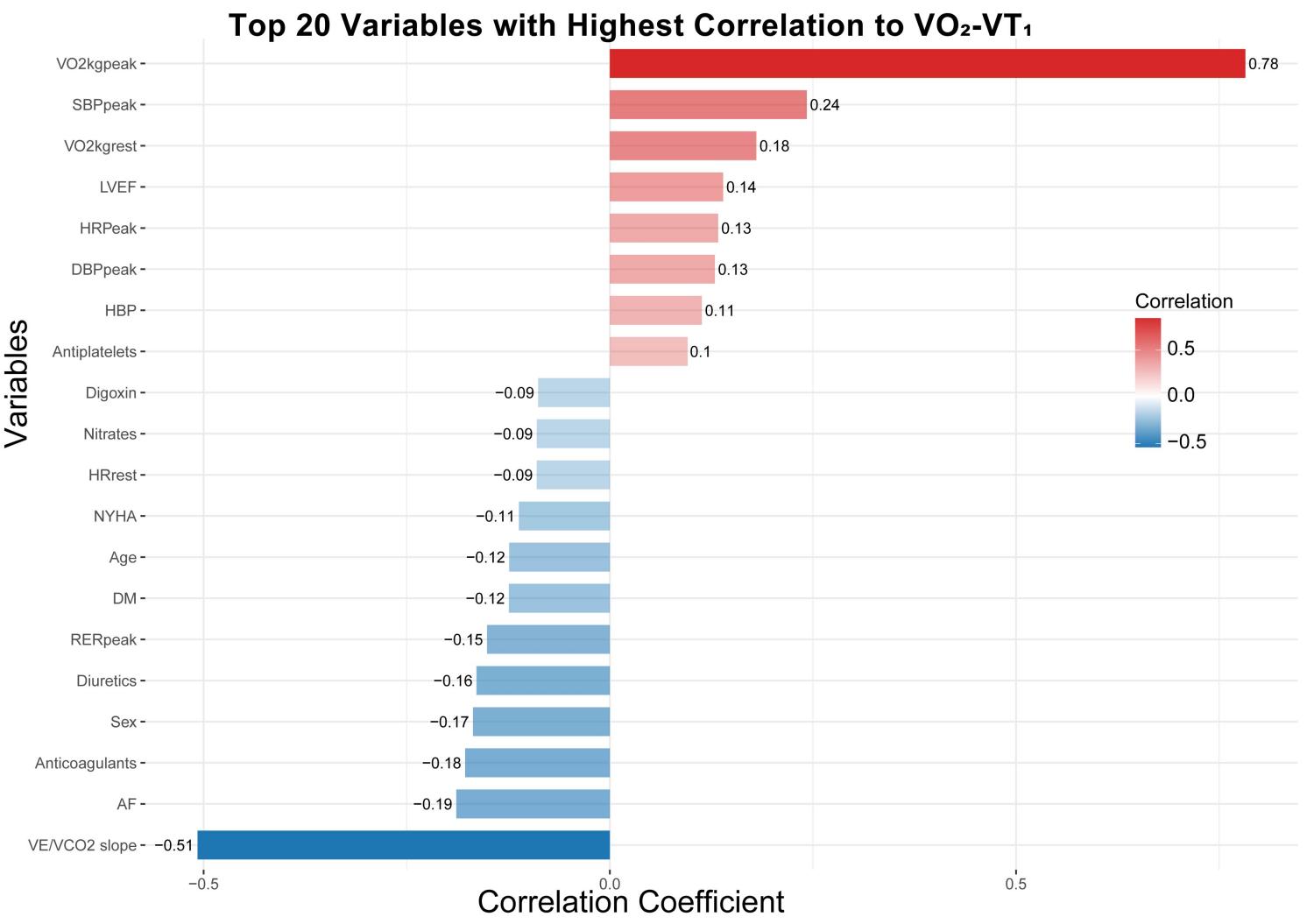
**

**Supplemental Figure 1.** Distribution of Top 20 Variables with Highest Correlation to VO2-VT_1_ in CHF Patients.

**
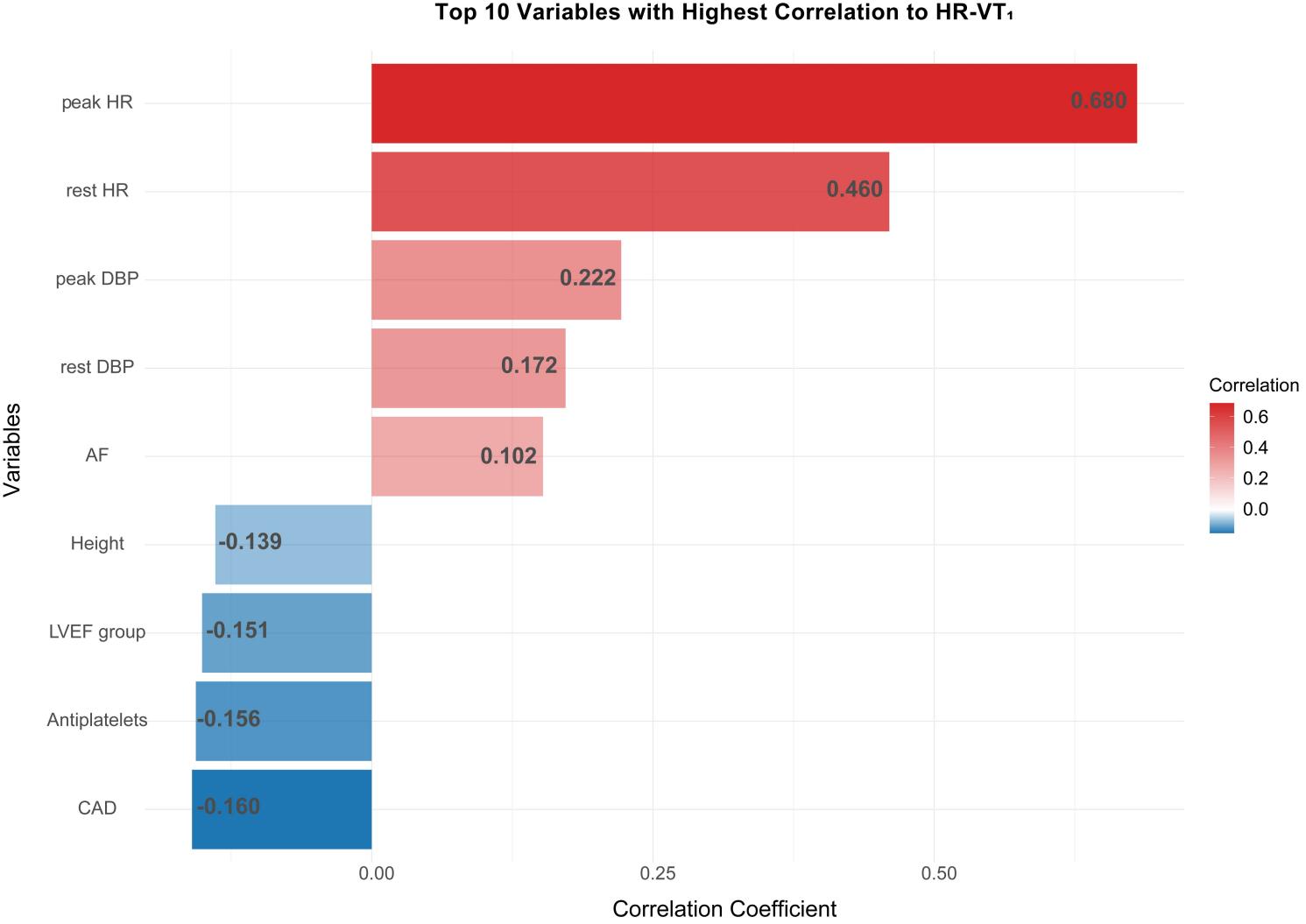
**

**Supplemental Figure 2.**  Distribution of Top 10 Variables with Highest Correlation to HR-VT_1_ in CHF Patients.

**
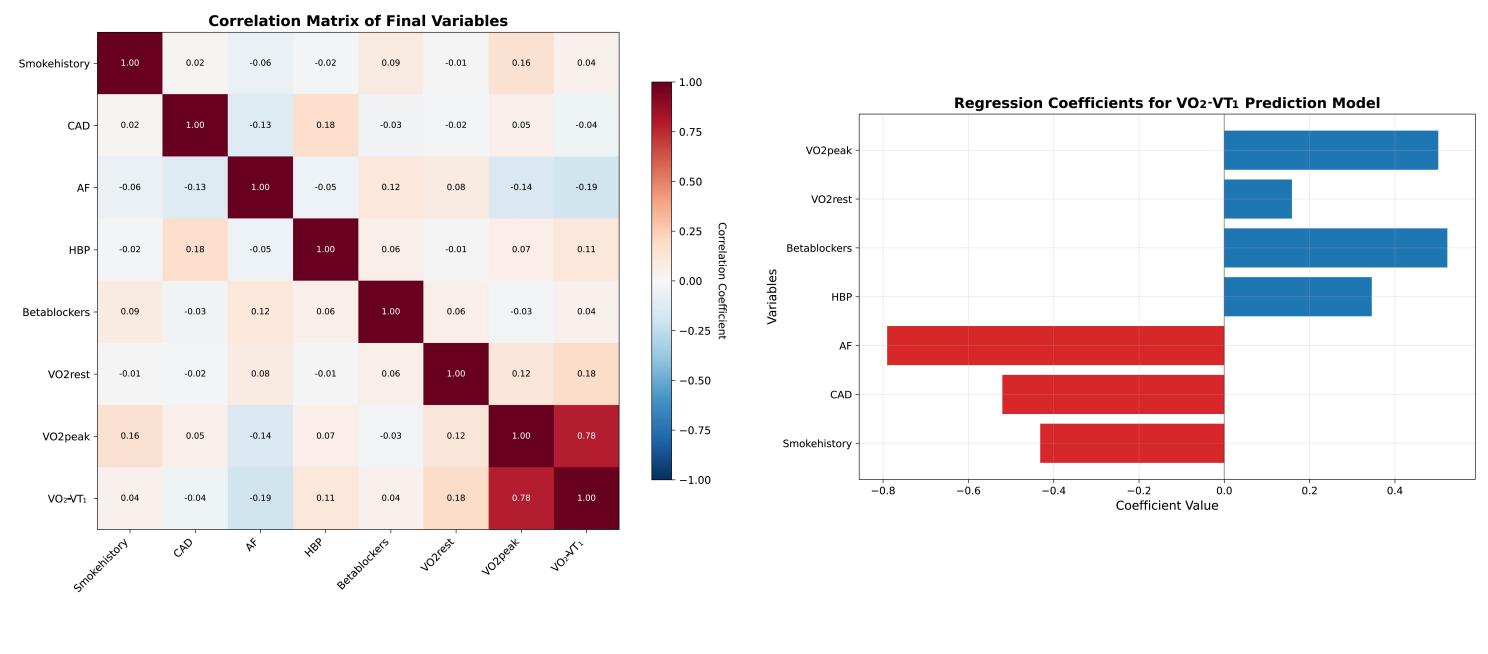
**

**Supplemental Figure 3.**  Correlation and regression coefficients for VO_2_-VT_1_ Prediction Model in CHF Patients.

**
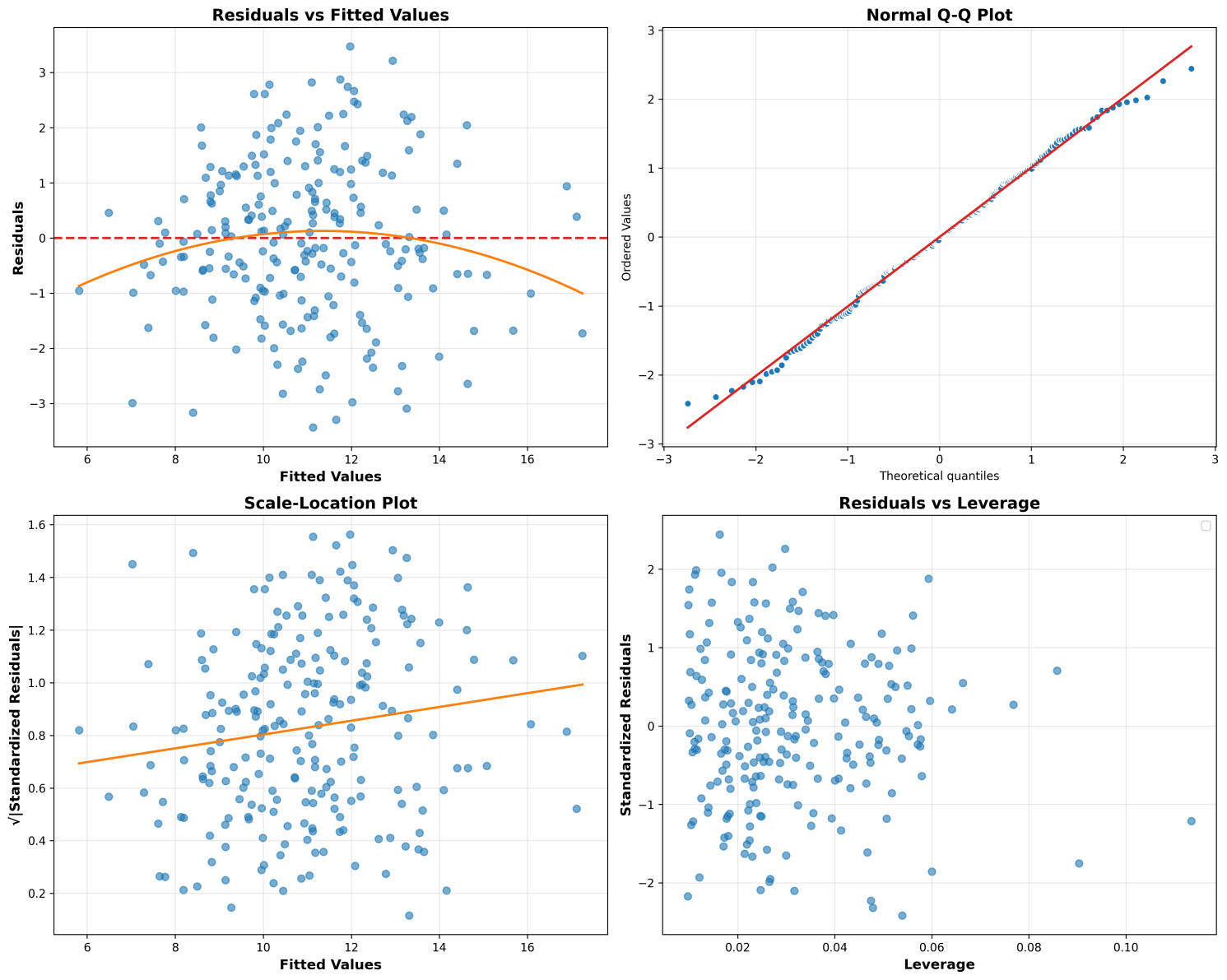
**

**Supplemental Figure 4.**  Residual Diagnostic Plots for VO2-VT_1_ Prediction Model in CHF Patients.

**
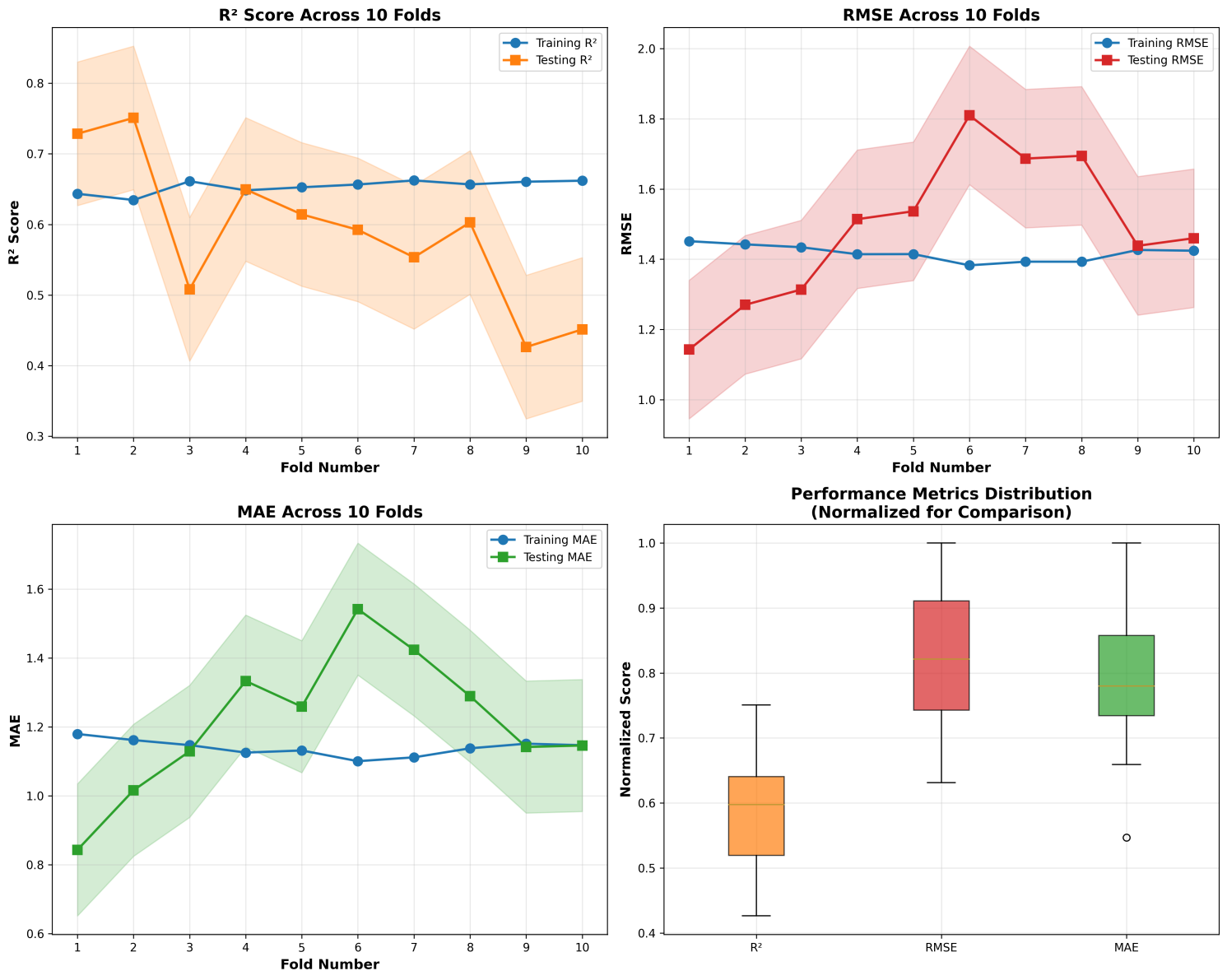
**

**Supplemental Figure 5.**  Cross Validation Performance for VO2-VT_1_ Prediction Model in CHF Patients.


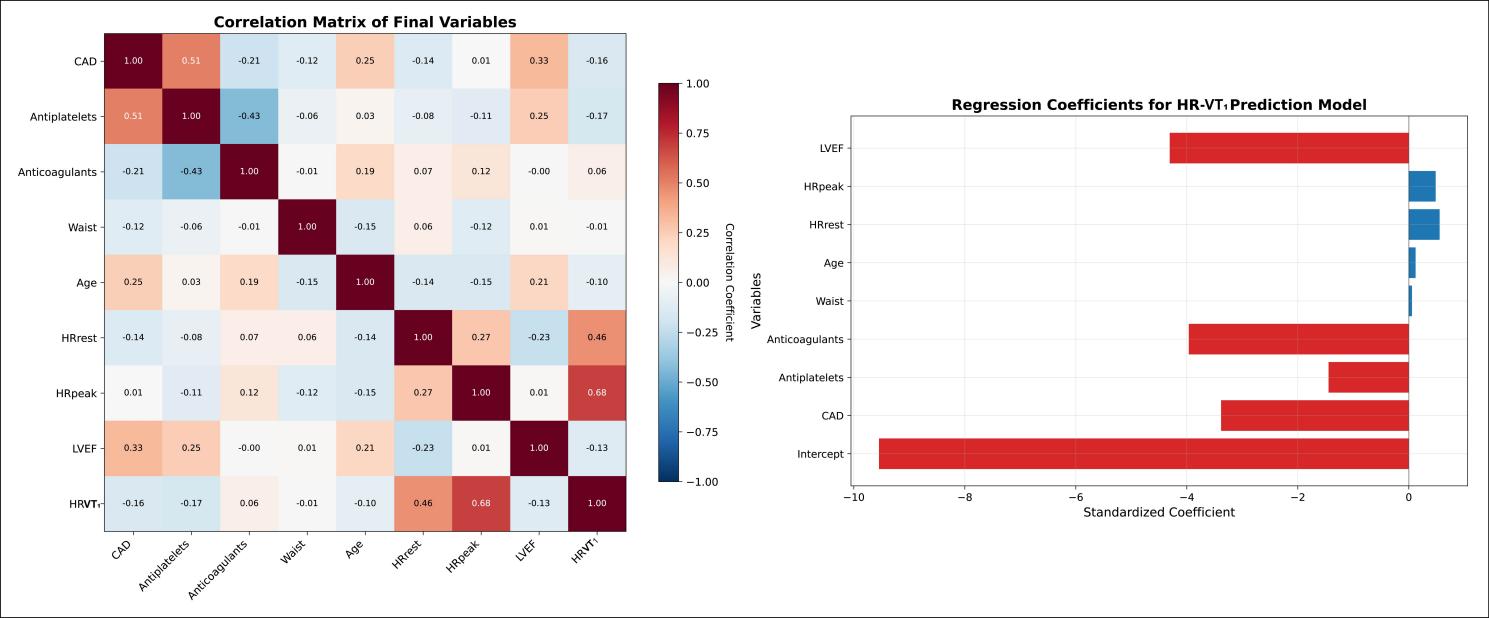


**Supplemental Figure 6.**  Correlation and regression coefficients for HR-VT_1_ Prediction Model in CHF Patients.

**
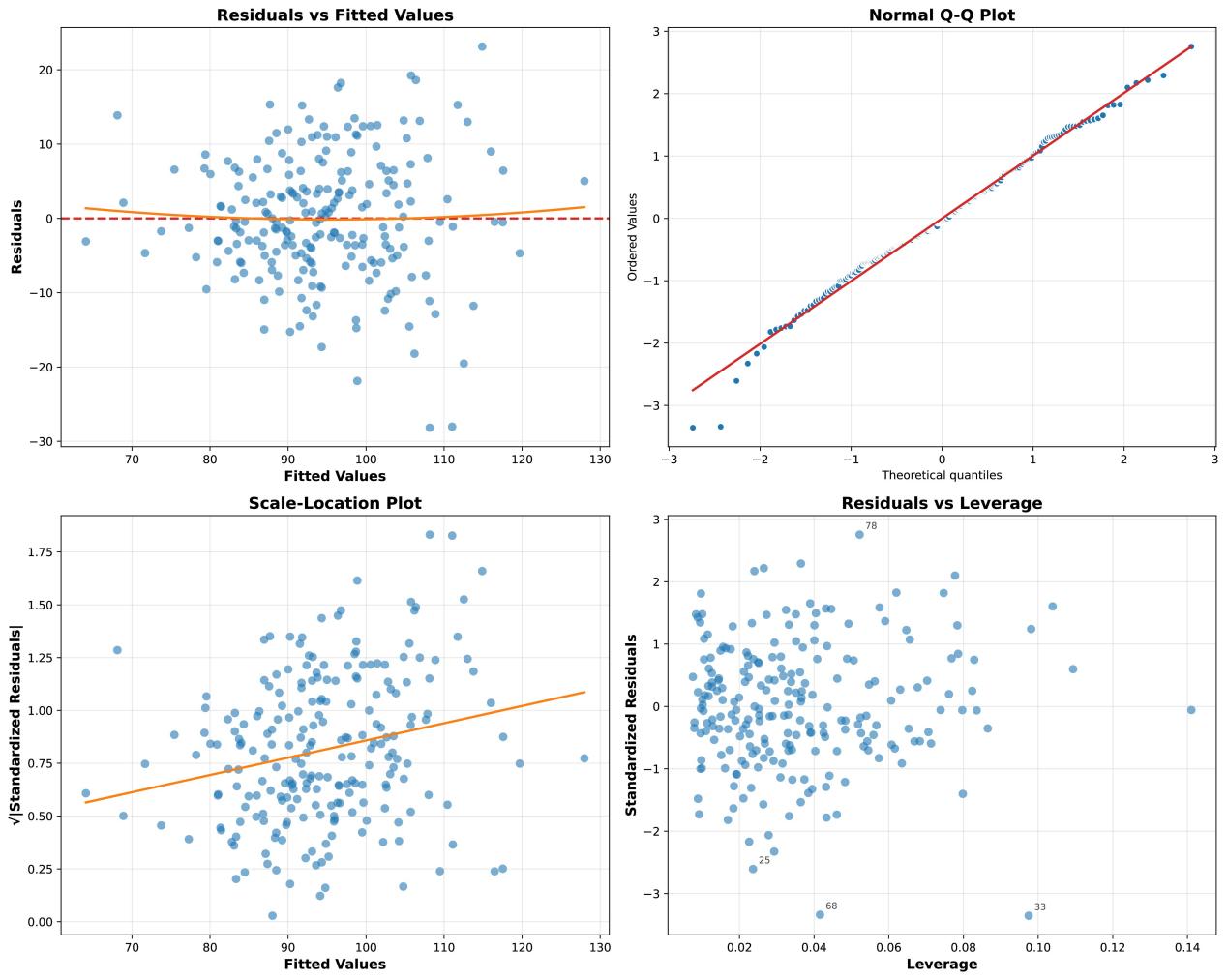
**

**Supplemental Figure 7.**  Residual Diagnostic Plots for HR-VT_1_ Prediction Model in CHF Patients.

**
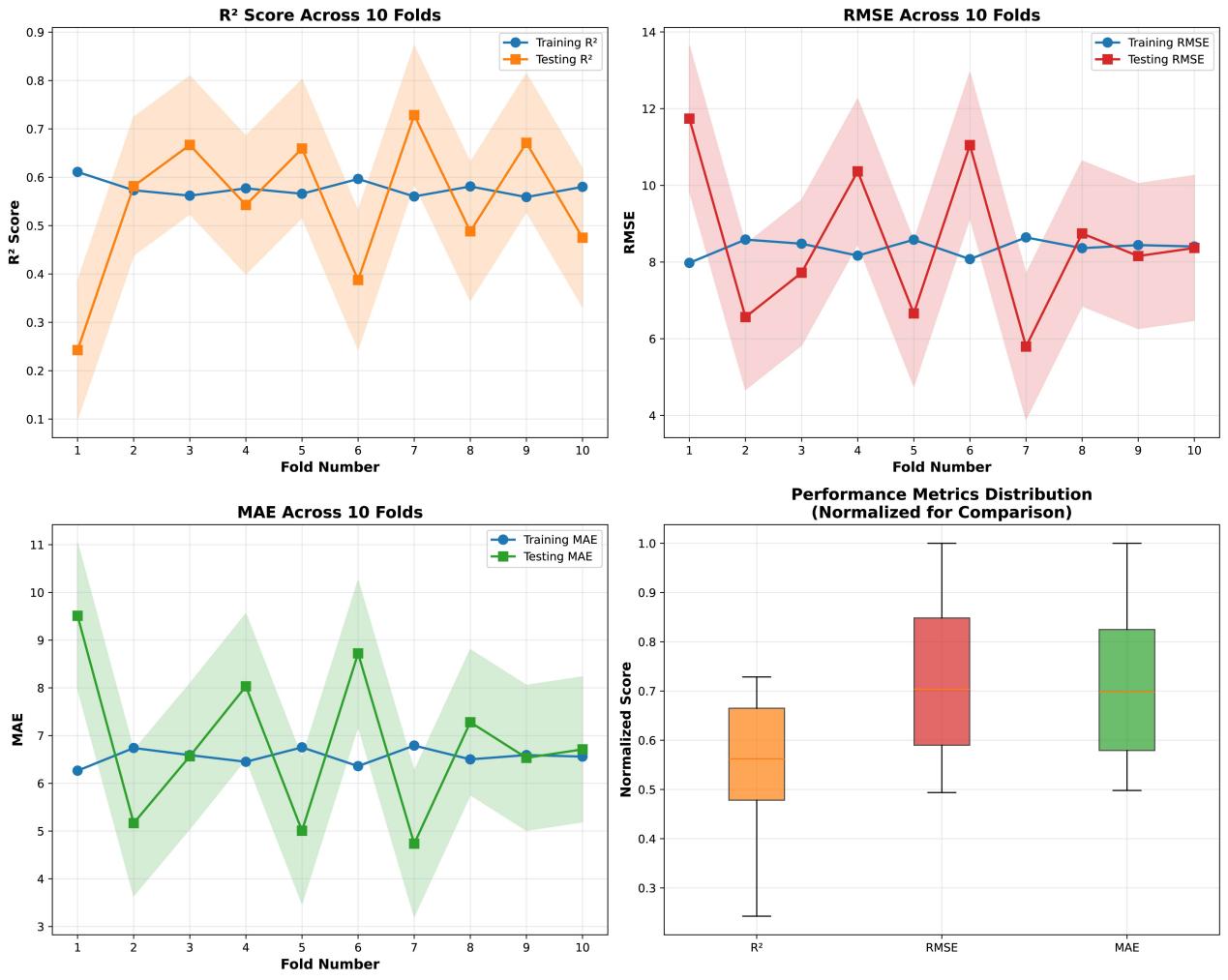
**

**Supplemental Figure 8.**  Cross Validation Performance for HR-VT_1_ Prediction Model in CHF Patients.

## Supplementary Tables

**Supplemental Table 1** Distribution of VO₂-VT_1_ as Percentage of Peak VO₂ in CHF Patients

| VO₂ -VT_1_ as Percentage of Peak VO₂ | Number of Patients, Proportion | Mean | Median |
| --- | --- | --- | --- |
| 30% - 40% | 0, 0% | - | - |
| 40% - 50% | 3, 1.3% | 47.8% | 48.9% |
| 50% - 60% | 23, 10.2% | 56.4% | 56.8% |
| 60% - 70% | 62, 27.6% | 65.3% | 65.9% |
| 70% - 80% | 71, 31.6% | 74.0% | 73.1% |
| 80% - 90% | 47, 20.9% | 84.5% | 83.7% |
| 90% - 100% | 19, 8.4% | 93.5% | 93.7% |

**Abbreviations**: VO₂-VT_1_: Oxygen Consumption at first ventilatory threshold .

**Supplemental Table 2** Distribution of HR-VT_1_ as Percentage of Peak HR in CHF Patients

| HR-VT_1_ as Percentage of Peak HR | Number of Patients, Proportion | Mean | Median |
| --- | --- | --- | --- |
| 30% - 40% | 0, 0% | - | - |
| 40% - 50% | 0, 0% | - | - |
| 50% - 60% | 2, 0.9% | 53.4% | 56.9% |
| 60% - 70% | 17, 7.6% | 62.3% | 64.5% |
| 70% - 80% | 75, 33.3% | 74.9% | 73.1% |
| 80% - 90% | 95, 42.2% | 83.8% | 84.0% |
| 90% - 100% | 36, 16.0% | 93.7% | 93.0% |

**Abbreviations**: HR-VT_1_: Heart Rate at first ventilatory threshold .

**Supplemental Table 3** Results of Pearson Correlation Analysis of VO₂-VT_1_ for Measurement Data

| Variables | Correlation Coefficients | P value |
| --- | --- | --- |
| peak VO₂ | 0.508 | P<0.001 |
| Weight | -0.003 | 0.853 |
| BSA | 0.143 | 0.885 |
| VE/VCO₂ slope | -0.139 | P<0.001 |
| BMI | -0.033 | 0.492 |
| Waist | -0.007 | 0.628 |
| rest VO₂ | 0.330 | 0.007 |
| Height | 0.014 | 0.552 |
| Waist-to-height ratio | -2.019 | 0.420 |
| peak SBP | 0.023 | P<0.001 |
| Age | -0.027 | 0.064 |
| peak DBP | 0.022 | 0.053 |
| peak RER | -7.127 | 0.023 |
| LVEF | 2.859 | 0.037 |
| rest DBP | -0.017 | 0.498 |
| rest SBP | 0.008 | 0.566 |
| peak HR | 0.019 | 0.046 |
| rest HR | -0.034 | 0.179 |

**Abbreviations**: VO₂: Oxygen Consumption; BSA: Body Surface Area; VE: Minute Ventilation; VCO₂: Carbon Dioxide Excretion; BMI: Body Mass Index; SBP: Systolic Blood Pressure; DBP: Diastolic Blood Pressure; RER: Respiratory Exchange Ratio; LVEF: Left Ventricular Ejection Fraction; HR: Heart Rate.

**Supplemental Table 4** Results of Spearman correlation analysis of VO₂-VT_1_ for categorical variables

| Variables | Correlation Coefficients | P value |
| --- | --- | --- |
| Sex | -1.052 | 0.011 |
| AF | -1.360 | 0.004 |
| HBP | 0.587 | 0.090 |
| ACEI/ARB | 0.161 | 0.678 |
| Anticoagulants | -1.321 | 0.007 |
| HBP classification | 0.993 | 0.297 |
| Diuretics | -0.829 | 0.014 |
| Smoke history | 0.210 | 0.528 |
| CAD | -0.207 | 0.547 |
| Nitrates | -0.501 | 0.179 |
| MI | -0.294 | 0.369 |
| Beta blockers | 0.309 | 0.509 |
| Type 2 diabetes | -0.670 | 0.063 |
| Antiplatelets | 0.525 | 0.152 |
| Hyperlipidaemia | 0.116 | 0.746 |
| Digoxin | -0.546 | 0.187 |
| PCI | 0.102 | 0.753 |
| Alcohol consumption history | -0.158 | 0.726 |

**Abbreviations**: AF: Atrial Fibrillation; HBP: High Blood Pressure; ACEI: Angiotensin-Converting Enzyme Inhibitor; ARB: Angiotensin Receptor Blocker; CAD: Coronary Artery Disease; MI: Myocardial Infarction; PCI: Percutaneous Coronary Intervention.

**Supplemental Table 5** Results of Pearson Correlation Analysis of HR-VT_1_ for Measurement Data

| Variables | Correlation Coefficients | P value |
| --- | --- | --- |
| peak HR | 0.680 | P<0.001 |
| rest HR | 0.460 | P<0.001 |
| peak DBP | 0.222 | P<0.001 |
| rest DBP | 0.172 | 0.010 |
| Height | -0.139 | 0.037 |
| LVEF | -0.127 | 0.057 |
| Age | -0.097 | 0.147 |
| BSA | -0.096 | 0.149 |
| VE/VCO₂ slope | 0.085 | 0.202 |
| rest VO₂ | 0.083 | 0.397 |
| peak VO₂ | -0.057 | 0.213 |
| Weight | -0.056 | 0.403 |
| peak SBP | 0.040 | 0.551 |
| Waist-to-height ratio | 0.037 | 0.578 |
| rest SBP | 0.013 | 0.848 |
| peak RER | -0.011 | 0.873 |
| BMI | 0.008 | 0.900 |
| Waist | -0.007 | 0.915 |

**Abbreviations**: HR: Heart Rate; DBP: Diastolic Blood Pressure; LVEF: Left Ventricular Ejection Fraction; BSA: Body Surface Area; VE: Minute Ventilation; VCO₂: Carbon Dioxide Excretion; VO₂: Oxygen Consumption; SBP: Systolic Blood Pressure; RER: Respiratory Exchange Ratio; BMI: Body Mass Index.

**Supplemental Table 6** Results of Spearman correlation analysis of HR-VT_1_ for categorical variables

| Variables | Correlation Coefficients | P value |
| --- | --- | --- |
| CAD | -0.160 | 0.016 |
| Antiplatelets | -0.156 | 0.019 |
| Hyperlipidaemia | -0.120 | 0.072 |
| Digoxin | 0.115 | 0.085 |
| AF | 0.102 | 0.128 |
| HBP classification | -0.098 | 0.145 |
| Diuretics | 0.082 | 0.219 |
| HBP | -0.078 | 0.245 |
| MI | -0.074 | 0.271 |
| Smoke history | -0.072 | 0.281 |
| Sex | 0.068 | 0.313 |
| PCI | -0.065 | 0.330 |
| ACEI/ARB | -0.054 | 0.422 |
| Anticoagulants | 0.049 | 0.462 |
| Nitrates | 0.030 | 0.658 |
| Type 2 diabetes | 0.029 | 0.667 |
| Alcohol consumption history | -0.022 | 0.738 |
| Beta blockers | -0.018 | 0.791 |

**Abbreviations**: CAD: Coronary Artery Disease; AF: Atrial Fibrillation; HBP: High Blood Pressure; MI: Myocardial Infarction; PCI: Percutaneous Coronary Intervention; ACEI: Angiotensin-Converting Enzyme Inhibitor; ARB: Angiotensin Receptor Blocker.

**Supplemental Table 7** Results of Multiple linear regression analysis of VO₂-VT_1_ for selected variables

| Variables | Regression Coefficients | Std. Error | t value | P value |
| --- | --- | --- | --- | --- |
| (Intercept) | 2.660 | 0.051 | 0.089 | 0.929 |
| Smoke history | -0.432 | 0.206 | -2.095 | 0.037* |
| CAD | -0.520 | 0.220 | -2.365 | 0.019* |
| AF | -0.790 | 0.293 | -2.698 | 0.007** |
| HBP | 0.346 | 0.212 | 1.632 | 0.104 |
| Beta blockers | 0.523 | 0.281 | 1.859 | 0.064 |
| rest VO₂ | 0.159 | 0.074 | 2.142 | 0.033* |
| peak VO₂ | 0.501 | 0.028 | 18.150 | <0.001*** |

**Abbreviations**: CAD: Coronary Artery Disease; AF: Atrial Fibrillation; HBP: High Blood Pressure; VO₂: Oxygen Consumption. ***: P < 0.001; **: P < 0.01; *: P < 0.05.

**Supplemental Table 8** Results of Multiple linear regression analysis of HR-VT_1_ for selected variables

| Variables | Regression Coefficients | Std. Error | t value | P value |
| --- | --- | --- | --- | --- |
| (Intercept) | -9.547 | 10.27663 | -0.929 | 0.3540 |
| CAD | -3.383 | 1.51235 | -2.237 | 0.0263* |
| Antiplatelets | -1.446 | 1.66783 | -0.867 | 0.3870 |
| Anticoagulants | -3.964 | 2.02910 | -1.954 | 0.0521 |
| Waist | 0.05721 | 0.05234 | 1.093 | 0.2755 |
| Age | 0.122 | 0.05775 | 2.113 | 0.0358* |
| rest HR | 0.555 | 0.09664 | 5.743 | 3.13e-08*** |
| peak HR | 0.485 | 0.03692 | 13.126 | <2e-16*** |
| LVEF | -4.308 | 5.39637 | -0.798 | 0.4256 |

**Abbreviations**: CAD: Coronary Artery Disease; HR: Heart Rate; LVEF: Left Ventricular Ejection Fraction. ***: P < 0.001; **: P < 0.01; *: P < 0.05.

**Supplemental Table 9** Descriptive statistics of VO₂-VT_1_, VO₂-VT_1__pred, HR-VT_1_, and HR-VT_1__pred

| Variable | N | Mean | SD | Min | Max | Median | IQR |
| --- | --- | --- | --- | --- | --- | --- | --- |
| VO₂-VT_1_ | 225 | 10.96 | 2.42 | 4.04 | 17.83 | 10.71 | 3.37 |
| VO₂-VT_1__pred | 225 | 10.96 | 1.95 | 5.82 | 17.24 | 10.93 | 2.32 |
| HR-VT_1_ | 225 | 94.60 | 12.87 | 61.00 | 138.00 | 94.00 | 16.00 |
| HR-VT_1__pred | 225 | 92.83 | 9.76 | 61.97 | 125.11 | 91.88 | 12.53 |

**Abbreviations:** VO₂: Oxygen Consumption; HR: Heart Rate; IQR: interquartile range.

**Supplemental Table 10** Comparative Analysis of Existing Studies and CHF-Specific Innovations

| Study | Population | Methodological limitations | Breakthroughs of this research |
| --- | --- | --- | --- |
| Pymer et al. (2020)(1) | 112 patients with coronary heart disease | Only describes the problem, no solution provided.  Not focused on patients with heart failure. | Develop the VO₂ Prediction Model and AT HR Prediction Model for CHF patients. |
| Hansen et al. (2019)(2) | 272 cardiovascular disease patients without pacemaker. | Only describes the problem, no solution provided.  Not focused on patients with heart failure. | Develop the VO₂ Prediction Model and AT HR Prediction Model for CHF patients. |
| Anselmi et al. (2021)(3) | 167 cardiac patients, 150 healthy sedentary subjects, and 33 competitive endurance athletes. | Not focused on patients with heart failure.  Unexplored prediction tools. | Develop the VO₂ Prediction Model and AT HR Prediction Model for CHF patients. |
| Sempere-Ruiz et al. (2025)(4) | 20 CHF patients. | The absence of a priori sample-size estimation, which affects power assessment. | Making an advance estimation of the sample size ensures the efficacy assessment. |

**Supplemental Table 11** Distribution of %HRR-VT_1_ in CHF Patients

| %HRR-VT_1_ | Number of Patients, Proportion | Mean | Median |
| --- | --- | --- | --- |
| 30%-40% | 34, 15.1% | 36.5 | 36.8 |
| 40%-50% | 44, 19.6% | 45 | 44.1 |
| 50%-60% | 44, 19.6% | 55.5 | 55.6 |
| 60%-70% | 33, 14.7% | 65.3 | 65.1 |
| 70%-80% | 17, 7.6% | 74.6 | 76.5 |
| 80%-90% | 8, 3.6% | 83.9 | 84.1 |
| 90%-100% | 6, 2.7% | 93.3 | 93.4 |

**References：**

1. Pymer S, Nichols S, Prosser J, Birkett S, Carroll S, Ingle L. Does Exercise Prescription Based on Estimated Heart Rate Training Zones Exceed the Ventilatory Anaerobic Threshold in Patients with Coronary Heart Disease Undergoing Usual-Care Cardiovascular Rehabilitation? A United Kingdom Perspective. *Eur J Prev Cardiol* (2020) 27(6):579-89. Epub 2019/05/23. doi: 10.1177/2047487319852711.

2. Hansen D, Bonné K, Alders T, Hermans A, Copermans K, Swinnen H, et al. Exercise Training Intensity Determination in Cardiovascular Rehabilitation: Should the Guidelines Be Reconsidered? *Eur J Prev Cardiol* (2019) 26(18):1921-8. Epub 2019/06/21. doi: 10.1177/2047487319859450.

3. Anselmi F, Cavigli L, Pagliaro A, Valente S, Valentini F, Cameli M, et al. The Importance of Ventilatory Thresholds to Define Aerobic Exercise Intensity in Cardiac Patients and Healthy Subjects. *Scand J Med Sci Sports* (2021) 31(9):1796-808. Epub 2021/06/26. doi: 10.1111/sms.14007.

4. Sempere-Ruiz N, Manresa-Rocamora A, Fuertes-Kenneally L, Sanz-Rocher A, Baladzhaeva S, Climent-Payá V, et al. Detection of Exercise Intensity Thresholds in Patients with Chronic Heart Failure Based on Correlation Properties of Heart Rate Variability. *European Journal of Applied Physiology* (2025) 125(12):3475-84. doi: 10.1007/s00421-025-05860-9.
